# Deletion of the *CYP2D6* gene as a likely explanation for the serious side effects of the antipsychotic drug pimozide: a case report

**DOI:** 10.3389/fphar.2023.1237446

**Published:** 2023-08-10

**Authors:** Fernando Facal, Begoña Portela, Almudena Gil-Rodríguez, Francisco Barros, Olalla Maroñas, Angel Carracedo

**Affiliations:** ^1^ Servizo de Psiquiatría, Complexo Hospitalario Universitario de Santiago de Compostela, Servizo Galego de Saúde (SERGAS), Santiago de Compostela, Galicia, Spain; ^2^ Grupo de Genética Psiquiátrica, Instituto de Investigación Sanitaria de Santiago de Compostela (IDIS), Santiago de Compostela, Spain; ^3^ Grupo de Genética, Instituto de Investigación Sanitaria de Santiago de Compostela (IDIS), Santiago de Compostela, Spain; ^4^ Grupo de Medicina Xenómica, Centro de Investigación en Medicina Molecular y Enfermedades Crónicas (CIMUS), Universidade de Santiago de Compostela (USC), Santiago de Compostela, Spain; ^5^ Centro de Investigación Biomédica en Red de Enfermedades Raras, Instituto de Salud Carlos III (CIBERER), Madrid, Spain; ^6^ Grupo de Medicina Xenómica, Fundación Pública Galega de Medicina Xenómica (FPGMX), Santiago de Compostela, Spain

**Keywords:** severe adverse reactions, pharmacogenetics, parkinsonism, extrapyramidal syndrome, risperidone, pimozide, transaminases

## Abstract

*CYP2D6* analysis prior to the prescription of pimozide is required above a certain dose by the Food and Drug Administration in order to detect individuals with the poor metabolizer status. This precautionary measure aims to prevent the occurrence of serious adverse drug reactions. This study presents a case of a patient diagnosed with schizophrenia spectrum disorder. The patient suffered re-admission in the psychiatry ward because of severe secondary symptoms due to the antipsychotic drug pimozide, previously prescribed on a first admission. In order to assess the patient’s medication profile, real-time PCR was performed to analyze the main genes responsible for its metabolization, namely, *CYP2D6* and *CYP3A4*. The pharmacogenetic study revealed that the patient is a poor metabolizer for *CYP2D6*, presenting deletion of both copies of the gene (diplotype *5/*5). Fortunately, the symptomatology disappeared after the withdrawal of the responsible drug. In conclusion, abiding by the pharmacogenetic clinical practice guidelines and the pharmacogenetic analysis of *CYP2D6* when prescribing pimozide would have probably saved the patient from the consequences of severe side effects and the health system expenditure. There is an important need for more training in the pharmacogenetic field for specialists in psychiatry.

## Introduction

Schizophrenia pharmacological treatment is based on antipsychotic drugs (Schneider-Thoma et al., 2022). The response to drugs is affected by genetic and non-genetic factors such as age, gender, renal and hepatic functions, concomitant treatments, and comorbid diseases, among others. Drug biotransformation is mediated by multiple genes. Differences in the variants present in these genes result in variations in the expected response to drugs; therefore, pharmacogenetics can be helpful in guiding drug prescription to minimize side effects or lack of efficacy (Arranz and de Leon, 2007; Yoshida and Müller, 2020).

The metabolization of psychiatric drugs is mediated by the family of cytochrome P450; *CYP2D6* and *CYP2C19* are the main genes responsible for metabolization, while others, such as *CYP3A4*, contribute to a lesser extent^1^. Variants in these genes can codify normal, reduced, or null enzyme activities (Gaedigk et al., 2008; Gaedigk et al., 2017; Gaedigk et al., 2018). Concerning the functionality of the variants observed, different phenotypes can be defined for a gene. In the case of *CYP2D6*, four main metabolizer phenotypes have been described: normal, intermediate, poor, and ultrarapid.

There are already recommendations promulgated by regulatory agencies such as the Food and Drug Administration (FDA) or the European Medicines Agency (EMA) concerning the analysis of concrete pharmacogenetic biomarkers when administering psychiatric drugs. Additionally, two private consortia in pharmacogenetics, the Clinical Pharmacogenetics Implementation Consortium (CPIC)^2^ and the Dutch Pharmacogenetics Working Group (DPWG)^3^, have also published recommendations. Drug label annotations encompass recommendations according to the importance of the pharmacogenetic biomarkers in the metabolization of a drug. Recommendations are cataloged as follows: obligatory or recommended—for variants that should be considered for analysis prior to prescription of a certain drug, i.e., pimozide^4^; actionable—for variants related to efficacy and/or toxicity of a drug that can be analyzed, i.e., aripiprazole^5^ (Ravyn et al., 2013; Jukic et al., 2019); and informative—if there are variants that affect the efficacy and/or toxicity of a drug but are still with no clinically associated significant effect, i.e., risperidone^6^.

Pimozide is the only antipsychotic that requires genetic testing prior to prescription according to regulatory agencies^7^. The drug is mainly metabolized by *CYP2D6* (Chapron et al., 2020). The FDA-approved drug label for pimozide states that *CYP2D6* genotyping should be performed at doses above 4 mg/day in adults. Additionally, the FDA recommends doses not exceeding 4 mg/day in adults that are poor *CYP2D6* metabolizers and not increasing the dose earlier than 14 days^8^. On the other hand, patients who are *CYP2D6* intermediate metabolizers should be given no more than 80% of the standard maximum dose of pimozide while patients who are *CYP2D6* poor metabolizers should be given no more than 50% of the standard maximum dose^9^.

A case that exemplifies the usefulness of analyzing pharmacogenetics in a patient with schizophrenia treated with pimozide is presented.

## Materials and methods

### Case presentation

A case of a 63-year-old male patient of European ancestry diagnosed with unspecified schizophrenia spectrum disorder (DSM-5 criteria) is presented. Until the age of 62, neither physical nor psychiatric illnesses had been diagnosed, and no medication had been prescribed. The patient is divorced and is living with two children in a Spanish rural area. The patient presented no family history of mental disorders. He has a primary school education, and he has never been employed. He never used tobacco nor consumed any other recreational drugs except occasional alcohol intake during weekends, which he abandoned 2 years ago. At the age of 62 (53 kg and 163 cm), the patient had a first hospitalization in the Psychiatry Service for psychotic symptoms and behavioral alterations. Chronic delusional ideas of harm and reference were described. He was diagnosed with unspecified schizophrenia spectrum disorder. Initially, risperidone 6 mg/day was prescribed, producing hypertransaminasemia as a side effect (GOT 130 IU/L and GPT 204 IU/L). After the withdrawal of risperidone, the levels of transaminases were normalized. Pimozide 4 mg/day was prescribed, and hospital discharge proceeded after 39 days of admission, with clinical improvement, emotional distancing from delusional ideas, and behavioral normalization. After hospital discharge, he maintained ambulatory follow-up in the Mental Health Unit, where quetiapine 50 mg/day was prescribed to treat insomnia.

**FIGURE 1:**
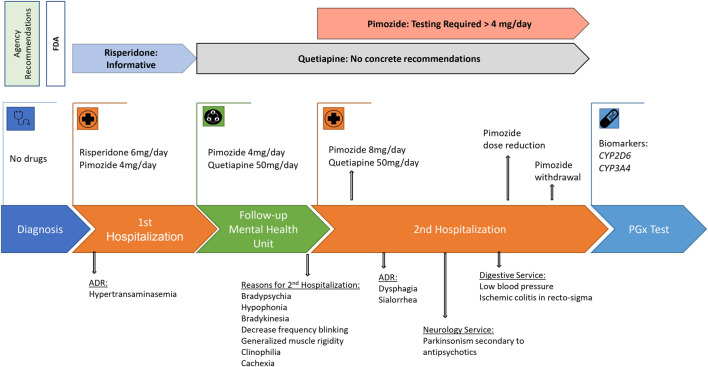
Clinical history of the patient and agency recommendations. PGx test, pharmacogenetics analysis; ADR, adverse drug reaction.

After 10 months of follow-up in the Mental Health Unit, the patient was hospitalized again in the Psychiatry Service for presenting a clinical worsening of months of evolution characterized by bradypsychia, hypophonia, bradykinesia, decrease in the frequency of blinking, generalized muscle rigidity, clinophilia, and loss of appetite and weight, reaching cachexia. An electrocardiogram showed a sinus rhythm at 68 beats per minute and a QTc of 394 ms. Initially, the case was oriented as a catatonic syndrome associated with the psychotic disorder, and the pimozide dose was increased to 8 mg/day. The symptoms worsened with dysphagia and sialorrhea, so the patient was evaluated by the Neurology Service that described bradykinesia and bilateral and symmetrical cogwheel rigidity and established the diagnostic hypothesis of parkinsonism secondary to antipsychotics. During this admission, the patient presented a tendency to low blood pressure and ischemic colitis in the recto-sigma that arose as a complication, so he was transferred to the Digestive Service. The dose of pimozide was progressively decreased until withdrawal. After this, parkinsonian symptomatology began to improve until complete resolution. During admission, no data of psychotic decompensation were objectified, despite quetiapine at a dose of 50 mg/day being the only antipsychotic treatment, so the hypothesis of catatonia was discarded (Figure 1). The causality of both drugs’ adverse reactions was defined with the Spanish Pharmacovigilance System (Table 1) (Aguirre and García, 2016). Due to the history of side effects with antipsychotics, a pharmacogenetic test was requested.

**TABLE 1:** Algorithm of causality assessment in reports of the Spanish Pharmacovigilance System (Aguirre and García, 2016).

Drug—adverse reaction/item in the algorithm	Score	Meaning	Observation
Risperidone—hypertransaminasemia			
1. Temporal sequence	2/2	Compatible	2 weeks after initiation of the drug: GOT 130 IU/L and GPT 204 IU/L. Not in a previous analysis just before the initiation: GOT 19 IU/L and GPT 11 IU/L
2. Previous knowledge	2/2	Described in the drug data sheet	Described in the drug data sheet as infrequent
3. Outcome after withdrawal	2/2	Improvement with withdrawal	2 weeks after withdrawal of the drug: GOT 30 IU/L and GPT 49 IU/L
4. Re-exposure	0/2	No re-exposure	-
5. Alternative causes	1/1	Sufficient information to rule out other causes	Hepatotropic virus serology negative. No alcohol consumption. No fever, no other symptoms, and no other alterations in laboratory tests
6. Contributing factors	1/1	Yes	*CYP2D6* poor metabolizer
7. Additional explorations	0/1	No	Drug plasma levels were not analyzed
Total score	8/11	Defined	-
			
Pimozide—parkinsonism			
1. Temporal sequence	2/2	Compatible	Onset some months after initiation of the drug. Rapid worsening after dose increase
2. Previous knowledge	2/2	Described in the drug data sheet	-
3. Outcome after withdrawal	2/2	Improvement with withdrawal	Rapid and progressive improvement after withdrawal
4. Re-exposure	0/2	No re-exposure	-
5. Alternative causes	1/1	Sufficient information to rule out other causes	Brain computed tomography and DAT-SCAN without alterations
6. Contributing factors	1/1	Yes	*CYP2D6* poor metabolizer
7. Additional explorations	0/1	No	Drug plasma levels were not analyzed
Total score	8/11	Defined	-

### Pharmacogenetic analysis

A pharmacogenetic test was requested to the Galician Public Foundation of Genomics Medicine (FPGMX)^10^ (Table 2). DNA isolation from blood was achieved using Chemagen technology (PerkinElmer). DNA concentration and purity measurements were performed using the Qubit^®^ 3.0 Fluorometer (Thermo Fisher Scientific) and NanoDrop (Thermo Fisher Scientific), respectively. TaqMan^®^ assays were used with a final reaction volume of 10 µL consisting of 5 µL of genotyping master mix, 0.5 µL of each assay probe, 2.5 µL of nuclease-free water, and finally, 2 µL of genomic DNA at a concentration of 2.5 ng/μL. PCR cycling conditions were as follows: a pre-read stage at 60°C for 30 s, a hold stage cycle at 95°C for 10 min, followed by 50 cycles of denaturation at 95°C for 15 s and annealing/extension at 60°C for 1 min 30 s, and finally, a post-read stage at 60°C for 30 s^11^.

**TABLE 2:** Pharmacogenetic results for the patient.

Gene	db ID	Genotype	Diplotype	Phenotype
*CYP2D6*	rs16947	NOAMP/NOAMP	*5/*5	PM
	rs1065852	NOAMP/NOAMP		
	rs28371725	NOAMP/NOAMP		
	rs35742686	NOAMP/NOAMP		
	rs3892097	NOAMP/NOAMP		
	rs5030655	NOAMP/NOAMP		
	rs5030656	NOAMP/NOAMP		
	Intron 2	0 copies		
	Intron 6	0 copies		
	Exon 9	0 copies		
*CYP2C19*	rs4244285	G/G	*1/*1	NM
	rs4986893	G/G		
	rs12248560	C/C		
*CYP3A4*	rs35599367	G/G	---	Absence of *22
*CYP1A2*	rs2069514	C/C	*1/*1	NM
	rs762551	T/T		

NM, normal metabolizer; PM, poor metabolizer; NOAMP: no amplification, which means that no alleles have been detected.

Copy number variation of *CYP2D6* was performed using a TaqMan^®^ assay mixture consisting of 5 µL of TaqPath ProAmp Master Mix, 0.5 µL of each assay probe, 0.5 µL of RNase P (as an endogenous reference control to normalization), 2 µL of nuclease-free water, and finally, 2 µL of genomic DNA at a concentration of 2.5 ng/μL. PCR cycling conditions were as follows: a hold stage compound by a step at 50°C for 2 min and a step at 95°C for 10 min, followed by 40 cycles of a step at 95°C for 15 s and a step at 60°C for 1 min. Moreover, negative and positive controls were used in the reactions. Technical replicates were used according to commercial protocol^12^.

Analyses were performed with real-time PCR in the QuantStudio™ 12K Flex instrument (Applied Biosystems by Life Technologies). For genotyping and copy number variation analysis, QuantStudio™ 12K Flex Real-Time PCR Software v1.4 and CopyCaller Software version 2.0 (Thermo Fisher Scientific) were used, respectively.

## Results

Pharmacogenetic results for the patient (Table 2) revealed compromised CYP2D6 enzyme activity with the diplotype of *CYP2D6* *5/*5. The no-functional CYP2D6*5 allele defines a complete deletion of the gene. In this case, the patient presents deletion of both copies of the *CYP2D6* gene. Based on the genotype, pharmacogenetic consortia categorize this patient as a poor metabolizer of CYP2D6 substrates, so there is a high risk for poor or adverse response to drugs metabolized by *CYP2D6*. Therefore, in order to avoid an adverse drug response, dose adjustments or alternative therapeutic agents may be necessary for drugs metabolized by *CYP2D6*. For *CYP3A4*, the patient does not present the *22 allele. It is worth highlighting that the allele CYP3A4*22 codifies for decreased activity of the enzyme.

## Discussion

In the current clinical case, the patient was sequentially treated with risperidone and pimozide and was reported to be a poor metabolizer of *CYP2D6*, the main gene responsible for the metabolism of both drugs. The patient suffered serious side effects, such as disabling parkinsonism with symptoms worsened by dysphagia and sialorrhea, once the pimozide dose was increased up to 8 mg/day. Additionally, the patient presented ischemic colitis that could be related to low blood pressure, possibly secondary to adverse reactions to pimozide. The patient presents deletion of both copies of the *CYP2D6* gene, so even low doses of antipsychotics could produce serious side effects. In this case, the FDA-approved drug label for pimozide recommends doses not exceeding 4 mg/day in adults^13^. Additionally, the DPWG provides dose reductions for pimozide up to 50% of the normal maximum dose^14^. Despite the FDA recommendations to perform only the analysis of *CYP2D6* while increasing doses of pimozide up to 4 mg/day, given the clinical characteristics of the case, it would have been very useful to perform a pharmacogenetic test when prescribing pimozide and definitely when increasing the dose up to 8 mg/day. In this concrete case, a pharmacogenetic analysis would have been helpful in the selection of antipsychotic treatment after the withdrawal of pimozide. The lack of a pharmacogenetic test would probably have led to the blind prescription of antipsychotics, which are probably metabolized by the *CYP2D6* pathway.

The percentage of the European population described to have a deletion of one copy of *CYP2D6* is approximately 3%–5% (Pratt et al., 2021). Thus, presenting deletion of both copies of the gene is unusual^15^ (Taylor et al., 2020). Additionally, this situation will directly affect the patient’s relatives since at least one non-functional copy is inherited. However, deletion of *CYP2D6* copies is not the only situation for a patient to be categorized as a poor metabolizer. There are multiple alleles in *CYP2D6* which codify for decreased or no functional activity of the enzyme. The frequency of poor metabolizers for *CYP2D6* in the European population has been reported to be approximately 7%^9^. In these patients, it is quite possible that even low doses of antipsychotics can produce serious adverse effects.

Pharmacogenetics is a feasible, ready-to-use tool in clinical practice that must be assessed by the prescribing physician as part of a whole, integrating and contextualizing the pharmacogenetic information with the possible pharmacological interactions and the clinical/pharmacological history of the patient. Additionally, as demonstrated, pharmacogenetics can help guide the clinician when serious adverse effects occur. In our opinion, if the pharmacogenetic analysis had been requested beforehand, it would have probably saved the patient from the consequences of serious side effects and the health system expenditure. Therefore, there are still several barriers that hinder the implementation of pharmacogenetics into clinical care. Different studies have addressed these barriers across different countries (Agúndez et al., 2012; Abou Diwan et al., 2019). It is important to note that, in recent years, a lot of effort has been made to advance our understanding of the genes and the development of recommendations or guidelines. Additionally, pharmacogenetic tests have become more accessible in terms of price and, in some countries such as Spain and concretely in Galicia, have been included in the healthcare portfolio (Decreto del DOG n^o^ 96 de 2014/5/21 - Xunta de Galicia, 2014; Decreto del DOG n^o^ 213 de 2011/11/8 - Xunta de Galicia, 2011).

Although these efforts have helped progress the field of pharmacogenetics, education of healthcare professionals is likely to pose an important barrier to its widespread use since most psychiatrists hardly have genetic lessons during their specialized training period (Nurnberger et al., 2018). It is important to translate to healthcare professionals the benefits of analyzing certain genes not only prior to prescribing certain medications but also when unexpected side effects related to a drug are observed. Teaching professionals how to search pharmacogenetic information would help make a prescription of drugs guided by the genotype.

## Conclusion

The case presented highlights the importance of pharmacogenetic analysis in clinical practice in psychiatry. In this particular case, the pharmacogenetic analysis was useful in selecting the antipsychotic of choice after withdrawal of pimozide; however, we consider that it could have been used prior to prescription, which could have led to an improvement in terms of medication-related side effects and subsequent consequences.

This situation highlights the fact that although pharmacogenetics is a feasible, ready-to-use tool in clinical practice, there are barriers that still need to be solved. One of them is the need to train psychiatrists in the pharmacogenetics of biomarkers with recommendations by regulatory agencies. Thus, the corresponding genetic analysis could be requested when useful, and the result could be integrated into the clinical–pharmacological history of the patient. Pharmacogenetics is a tool to be used in conjunction with other clinical parameters that can help the clinician to personalize treatments. Finally, we highlight the importance of teaching professionals about pharmacogenetics, which may help them to make a prescription of drugs guided by the genotype.

## Data Availability

The original contributions presented in the study are included in the article/Supplementary Materials, further inquiries can be directed to the corresponding author.

## Footnotes

1
https://www.pharmgkb.org/

2
https://cpicpgx.org/

3
https://www.knmp.nl/dossiers/farmacogenetica

4
https://www.pharmgkb.org/chemical/PA450965/labelAnnotation

5
https://www.pharmgkb.org/chemical/PA10026/labelAnnotation

6
https://www.pharmgkb.org/chemical/PA451257/labelAnnotation

7
https://www.pharmgkb.org/labelAnnotation/PA166104870

8
https://www.pharmgkb.org/labelAnnotation/PA166104870

9
https://www.pharmgkb.org/chemical/PA450965/guidelineAnnotation/PA166182819

10
https://xenomica.sergas.gal/?idioma=es

11
https://assets.thermofisher.com/TFS-Assets/LSG/manuals/MAN0009593_TaqManSNP_UG.pdf

12
https://assets.thermofisher.com/TFS-Assets/LSG/manuals/4397425_CopyNumAssays_UG.pdf

13
https://www.pharmgkb.org/labelAnnotation/PA166104870

14
https://www.pharmgkb.org/chemical/PA450965/guidelineAnnotation/PA166182819

15
https://www.pharmgkb.org/page/cyp2d6RefMaterials
